# Assessing factors influencing adolescent sexual debut in Nigeria: a multi-cluster survival analysis approach

**DOI:** 10.3389/frph.2025.1475421

**Published:** 2025-01-23

**Authors:** Fabio Mathias Correa, Peter Enesi Omaku, Joseph Odunayo Braimah

**Affiliations:** ^1^ Department of Mathematical Statistics and Actuarial Sciences, University of the Free State, Bloemfontein, South Africa; ^2^ Department of Mathematics and Statistics, Federal Polytechnic Nasarawa, Nasarawa State, Nigeria; ^3^ Department of Mathematics and Statistics, Ambrose Alli University, Ekpoma, Edo State, Nigeria

**Keywords:** generalised additive model, Besag, conditional auto-regressive, INLA, spatial analysis

## Abstract

**Introduction:**

Early sexual debut is an area of concern in Nigeria with implications for reproductive health.

**Methods:**

This study addresses this by proposing a more effective survival model—one that incorporates both independent and identically distributed (IID) and Besag intrinsically conditional auto-regressive (ICAR) random effect priors, using a generalised additive model that accounts for both individual and spatial influences on age at first sex. We analyse data from the 2018 Nigerian NDHS survey and compare our model with existing models: a model without the cluster frailty effect, a model that ignores the Besag ICAR and includes the IID, and a model that ignores the IID and includes only the Besag ICAR.

**Results:**

Our approach, which combines independent and spatial random effects, outperforms others based on statistical criteria (Deviation Information Criterion and the Widely Applicable Information Criterion).

**Discussion:**

As shown in this study, the proposed model effectively captures the complexity of age at first sex. In addition, visualisations reveal significant geographic and social clusters with high rates of early sexual debut in Nigeria. These findings highlight the importance of considering multi-level clustering to better understand and promote healthy sexual behaviour among young Nigerians through targeted interventions.

## 1 Introduction

Countries with a large adolescent population face numerous challenges. These include a heightened demand for resources in areas such as education, healthcare, and job training, which can strain government budgets. High youth unemployment rates often accompany a large adolescent population, leading to poverty and potentially social unrest. Furthermore, a dissatisfied youth population can be more susceptible to political instability.

Reproductive health issues are crucial to address for several reasons. Access to reproductive healthcare empowers individuals to make informed choices about their sexual and reproductive health, leading to improved overall well-being. Unintended pregnancies and unsafe abortions have significant economic consequences for individuals, families, and entire countries. Addressing reproductive health concerns also promotes gender equality and empowers women and girls. Finally, reproductive health issues have significant public health implications, including the spread of sexually transmitted infections and maternal mortality.

Sexual debut refers to the first instance of sexual intercourse. It is a significant milestone in an individual’s life that is often influenced by various factors, including peer pressure, family dynamics, socioeconomic status, cultural norms, and exposure to sexual content. Early sexual debut, especially before the age of 15, has been associated with an increased risk of sexually transmitted infections, unintended pregnancy, and emotional distress. It can also influence later sexual behaviors, potentially leading to risky sexual practices and difficulties in forming healthy relationships.

With one in four people in Nigeria aged between 10 and 19 years old and a prevalence of adolescent pregnancy of 19%, the country has a large adolescent population (1). Despite constituting a large and significant proportion of Nigeria’s population, the adolescent population has several sexual and reproductive health concerns (2). Fear of first sexual experience is significantly influenced by changes in preferences, experiences, desires, behaviour, and sexuality that occur during adolescence. These changes are fuelled by the fact that young people are growing up in a world very different from that of their parents (1).

Exposure to the internet and a wide range of information shapes their views on sexuality—the interaction of all these elements either directly or indirectly influences the choices and health of these children (3). Data from many countries, including Nigeria, show that sexual behaviour among adolescents has changed recently, as evidenced by the age of sexual debut. Many young people are experiencing early physical maturation and sexual debut (4–7). These transitions also affect young people’s sexual behaviour. For example, they can encourage risky sexual behaviour such as having multiple sexual partners (MSPs), which exposes young people to harmful sexual and reproductive health outcomes such as unwanted pregnancies and sexually transmitted infections (STIs). This risk is exacerbated when young people lack access to information about HIV/AIDS and other STIs, as well as the contraceptives they need to protect themselves (8–11).

Using the Cox proportional hazards model and Kaplan-Meier plot to examine factors associated with Age at First Sexual Debut (AFSD) a small percentage of respondents began having sex at age eight, and approximately 54.4% of respondents did so before age seventeen (12). The median age of first sexual experience was 16 years, meaning that over 50% of respondents had their first sex at or before the age of 16. The age of the first sexual encounter was strongly influenced by education, religion, geography, and place of residence. The work of (13) uses a two-stage mixed-effects parametric survival model with Weibull distribution. The model was used to analyse the association between community factors and age of sexual debut. The authors found that the risk of sexual debut among teenage girls was lower in communities that were wealthier and more ethnically diverse. They also found that women who married during the study period had sexual debut earlier than single women. Their results, disaggregated by marital status, also showed that higher community levels of female employment and female education were associated with a higher risk of sexual debut among unmarried adolescent girls, but not among married adolescent girls (13). Some authors agree (14–18) that early marriage, lack of education and poor family background in rural areas were associated with a higher likelihood of early sexual debut among women. Adolescents and young adults are in urgent need of sexual and reproductive health education to delay sexual debut, although their paths to this point are varied. One of the few studies conducted in sub-Saharan Africa (19) estimated the rate of sexual debut by sex, age, time and nation using a log-skewed logistic distribution to characterise the time to AFSD in a Bayesian spatio-temporal model. By comparing AFSD reported by the same birth cohorts in multiple survey rounds, they were able to statistically correct for reporting bias by sex and nation. The results showed that in 2015, the median AFSD ranged from 15.8 for women in Angola to 25.3 for men in Niger. In 37 out of 40 countries, AFSD was younger for women than for men. The difference was negligible in southern African countries and highest in Sahel countries.

Many studies have examined the social, familial, and demographic variables associated with sexual initiation and the causes of adolescents’ first consensual sexual encounters. However, little is known about how more recent contextual factors, such as the increase in “internet use,” the evolving social media space, which may influence the overall modelling outcome, and geographical elements, relate to AFSD. In order to investigate the relationship between AFSD and environmental factors, more relevant and modern multi-clustering modelling techniques have been proposed in this study. It is essential to analyse the effects of unobserved heterogeneity in the number of households and geographical variation in the age of sexual onset. Firstly, this type of approach is in line with the Nigerian National Health Plan, which aims to reduce and eventually eliminate health inequalities between socio-economic, racial/ethnic, gender and geographical categories.

The present study analyses the Age of Household Head (AHH), Sex of Household Head (SHH), Ever Married (EM), Recent Sexual Activity (RSA), Type of Residence (TPR), Education, Religion, Wealth Index (WID) as demographic covariates, Use of Internet (UI) as social variable, Household Number (HHN) was considered for clustering effect, Region and States were considered for geo-reference variables, while events were observed for Age at First Sexual Initiation (AFSI) for subjects less than 18 years, the work also considered and assessed the influence of a combo cluster (multi-cluster) information—(household numbers and state of origin) using the Integrated Nested Laplace Approximation (INLA) via Bayesian inference. Geographical analysis should help to identify those states and areas of the nation where a comparatively high percentage of subjects are engaged in high-risk sexual behaviour. This information could be used to develop and implement more effective intervention programmes tailored to the specific needs of different geographical areas. Four main objectives were therefore defined. First, to propose a new multi-cluster model to analyse the factors influencing Age at First Sexual Debut (AFSD) in Nigeria. Second, to compare the performance of this new model with existing models used to analyse AFSD in Nigeria in order to identify the most effective approach. Third, use a large and nationally representative sample to estimate the relative influence of different risk factors contributing to early first sexual debut among Nigerian adolescents under the age of 18. Finally, to provide a visual representation on a map of Nigeria showing the average impact of these determinants of AFSD across the country.

## 2 Methodology

Survey data for 127,546 respondents representing factors influencing AFSD across the country were collected from the 2018 Nigeria Demographic and Health Survey (NDHS). According to the 2018 NDHS, the median age at first sexual intercourse among women aged 20–49 years was about 17.2 years. In this study, AFSD was considered as time, and an event is observed if AFSD≤17 years, AFSD beyond 17 years was considered censored.

Statistical analyses were performed using R software.

### 2.1 Variable classification

The selection of variables for this study using data from the Nigeria Demographic and Health Survey (NDHS) is critical to ensuring the relevance of the research. Variables are selected based on their relevance to the hypothesis being investigated. Researchers typically focus on a subset of variables that are most relevant to the research objectives.

For the purpose of analysis, the age of the household head (AHH) was kept in its metric form, while the other factors were categorical; the sex of the household head (SHH) was coded 2 for male and 1 for female (reference category), ever been married (EM) was coded 0, 1, 2 for “no,” “former,” “married” respectively. Recent sexual activity (RSA) was coded 0, 1, 2, 3 for 0 “never had sex,” “active in the last 4 weeks,” “not active in the last 4 weeks—postpartum abstinence,” “not active in the last 4 weeks—not postpartum abstinence,” respectively. The highest level of education (HEL) was coded 0,1,2,3 for those with “no education,” “primary education,” “secondary education,” and “higher education” respectively. Internet Use (IU) was coded 0,1,2,3 for those with “never,” “yes, in the last 12 months,” “yes, before the last 12 months,” and “yes, can’t say when.” WID was coded 1, 2, 3, 4, 5 for those who were “poorest” (reference category), “poorer,” “middle,” “richer,” and “richest” respectively. Religion was coded 1, 2, 3, 4, 5 for those who were ’Catholic’ (reference category), “Other Christian,” “Islam,” “Traditionalist,” and “Other” respectively. Type of place of residence (TPR) was coded 2 for urban settlers and 1 for rural settlers (reference category). The region was coded 1, 2, 3, 4, 5, and 6 for persons residing in “North Central,” “North-East,” “North-West,” “South-East,” “South-South,” and “South-West” respectively. Table 1 presents data on 127,546 respondents from the 2018 Nigeria Demographic and Health Survey (NDHS).

**Table 1:** Descriptive statistics for age at first sexual debut in Nigeria.

Fixed effects	Classification	Status	
		AFSD>17/0	AFSD<=17/1
AHH	Min/Max	14/98	15/98
	Mean/Std. Dev.	46.54/11.64	46.52/12.99
SHH	Female	30,085 (83.18%)	81,541 (89.24%)
	Male	6,085 (16.82%)	9,834 (10.76%)
Ever married	Not married	532 (1.47%)	1,049 (1.15%)
	Formerly	35,421 (97.93%)	89,924 (98.41%)
	Married	217 (0.60%)	402 (0.44%)
RSA	No sexual activity	24,286 (67.14%)	67,910 (74.32%)
	Not postpartum	3,085 (8.53%)	5,930 (6.49%)
	Postpartum	8,799 (24.33%)	17,535 (19.19%)
Education	No education	9,111 (25.19%)	54,588 (59.74%)
	Primary	7,019 (19.41%)	18,292(20.02%)
	Secondary	14,599 (40.36%)	16,157 (17.68%)
	Higher	5,441 (15.04%)	2,338 (2.56%)
Internet use	No	30,655 (84.75%)	88,540 (96.90%)
	Yes, in the last 12 months	4,863 (13.44%)	2,357 (2.58%)
	Yes, before the last 12 months	652 (1.80%)	478 (0.52%)
Wealth index	Poorest	3,944 (10.90%)	27,204 (29.77%)
	Poorer	5,679 (15.70%)	23,769 (26.01%)
	Middle	7,687 (21.25%)	19,433 (21.27%)
	Richer	9,342 (25.83%)	13,868 (15.18%)
	Richest	9,518 (26.31%)	7,101 (7.77%)
Religion	Catholics	4,941 (13.66%)	6,194 (6.78%)
	Other christians	16,605 (45.91%)	22,711 (24.85%)
	Islam	14,351 (39.68%)	61,591 (67.40%)
	Traditionalist	182 (0.50%)	495 (0.54%)
	Others	91 (0.25%)	384 (0.42%)
TPR	Rural	18,468 (51.06%)	25,643 (28.06%)
	Urban	17,702 (48.94%)	65,732 (71.94%)
Region	NC	7,310 (20.21%)	14,346 (15.70%)
	NE	4,867 (13.46%)	21,426 (23.45%)
	NW	5,406 (14.95%)	34,522 (37.78%)
	SE	7,355 (20.33%)	6,717 (7.35%)
	SS	4,140 (11.45%)	8,296 (9.08%)
	SW	7,092 (19.61%)	6,068 (6.64%)

### 2.2 The Weibull distribution

Both AFT and PH models are used to parameterise Weibull distributions. The Weibull distribution works well when modelling data with monotone hazard rates that rise or fall exponentially with time (20). The scale parameter for the Weibull distribution is λ, the shape parameter is k, and the probability distribution function of the regression, survivor and hazard function is given by:

(1) $$f(t;k,\lambda )={\frac{kt}{\lambda }}^{k-1}\;e^{{\left(-\frac{t}{\lambda }\right)}^{k}}\;e^{\left(\vec{\beta }\vec{x}\right)},\;k,\lambda >0$$

(2) $$S(t)=e^{{\left(-\frac{t}{\lambda }\right)}^{k}}\;e^{\left(\vec{\beta }\vec{x}\right)}$$

and

(3) $$h(t)=h(t;k,\lambda )={\frac{kt}{\lambda }}^{k-1}{\left(\frac{t}{\lambda }\right)}^{k}\;e^{\left(\vec{\beta }\vec{x}\right)}$$

where $\vec{\beta }$ and $\vec{x}$ are vectors of coefficient and covariate respectively.

In an Accelerated Failure Time (AFT) model, the Weibull distribution is often used to directly model the survival time rather than the hazard function. The AFT model assumes that the log transformation of the survival time is linearly related to the covariates, this relationship allows the Weibull distribution to effectively model varying hazard rates over time within the AFT framework (21).

Incorporating the Weibull distribution into an AFT survival model, given a set of covariates $x_{i}$ for individual i, the AFT model can be expressed as:

(4) $$log(T_{i})=\beta^{T}x_{i}+\rho_{i}$$

where $T_{i}$ is the survival time for individual I, β is the vector of regression coefficients corresponding to the covariates $x_{i}$, and $\rho_{i}$ is a random error term assumed to follow a local scale distribution.

The survival time T can be expressed for the Weibull distribution as:

(5) $$T=k(-\log(U){)}^{\frac{1}{\lambda }}$$

where U is a uniformly distributed random variable between 0 and 1. In the AFT framework, the log-transformed survival time is:

(6) $$log(T)=log(K)+\frac{1}{\lambda }log(-log(U))$$

The AFT model with the Weibull distribution can be written as follows:

(7) $$log(T_{i})=\beta^{T}x_{i}+\sigma W$$

where W is modelled using an extreme value distribution due to the fact that the logarithm of the Weibull distribution follows an extreme value distribution, the Weibull distribution is employed here because it is a more flexible and versatile distribution that can model various survival patterns, including monotonic and non-monotonic rates.

From this equation, we can solve for the parameters β, λ and σ using Maximum Likelihood Estimation (MLE). The likelihood is constructed based on the survival times and censoring information. Once we have estimated β, we can use them to predict survival times for new observations based on their covariates. This formulation allows us to incorporate the Weibulldistribution into an AFT survival model, which provides a flexible framework for modelling survival times while accounting for the effects of covariates (22). In the AFT regression models, the effect of covariates determines the time scale in such a way that if $e^{\left(x^{\prime }\beta \right)}>1$, the effect of the covariate vector x is to decrease the survival process, and if $e^{\left(x^{\prime }\beta \right)}<1$, the effect is to increase the survival process (22).

### 2.3 The generalised additive models

The Generalised Additive Models (GAMS) are statistical models in which conventional multiple linear regression is generalised to allow for a much larger class of time variants and a non-linear functional form of the continuous covariates and their effects. GAMs, derived from the work of (20) as in:

(8) $$\eta (t;X,z,w)=f_{0}(t)+f(w_{i})+x^{\prime }\gamma$$

The function $f_{0}(t)$ is the baseline function, γ denotes the typical linear part of the predictor, and $f(w_{i})$ is the effect of the functional form of the metric covariate. A further extension in (23) applied generalised additive models to explore models with continuous functional forms for unobserved heterogeneity in clustered survival and competing risks data.

(9) $$\eta (t;X,w)=f_{0}(t)+x^{\prime }\gamma +f(w_{i})+v_{i}$$

where $v_{\text{i}}$ is unobserved heterogeneity for cluster effects.

GAMs provide a flexible and effective means of moving out of the “linear rut” in which a significant proportion of biostatistical modelling still resides (24). Used by (25) to propose a flexible continuous-time geoadditive survival model in the context of the GAMs cited by (22) and (26) to examine geographical effects together with potentially non-linear effects of other covariates.

(10) $$\eta (t;X,w,S)=f_{0}(t)+f(w_{i})+x^{\prime }\gamma +f_{spat}(s_{i})$$

In dealing with the linear rut of (17, 27, 28), and more recently (29), it has been shown how survival data with a time-varying functional form can be modelled in an incredibly flexible way by taking advantage of sophisticated inference techniques designed for generalised additive mixed models.

(11) $$\eta (t;z,X,w,S)=f_{0}(t)+f(z_{i})+x^{\prime }\gamma +f(w_{i})+v_{i}$$

where $f(z_{i})$ is the functional form of time varying covariate.

For the purposes of this study, the following GAM representation is proposed.

(12) $$\eta (t;X,w,v,S)=f_{0}(t)+x^{\prime }\gamma +f(w_{i})+v_{iid}+f_{B.ICAR}(S_{i})$$

The function $f_{0}(t)$ is the lognormal baseline function, γ denotes the typical linear part of the predictor, $f_{B.ICAR}(s_{i})$ represents a structured geographical effect driven by an intrinsic conditional autoregressive (ICAR) or Besag-ICAR prior for map visualisation, and $v_{\mathrm{iid}}$ is the random clustering or frailty effect term following an independently and identically distributed Gaussian prior. Where $v_{iid}+f_{B.ICAR}(s_{i})$ provides the combo clustering effect needed to improve the model. All previous survival analysis models have used Markov Chain Multi-Carlo (MCMC) techniques to estimate their parameters, but this study proposes the use of the Integrated Nested Laplace Approximation (INLA) in R for computational efficiency, accurate approximation and easy implementation of our complex Bayesian model (30).

### 2.4 Besag intrinsic conditional autoregressive prior

A conditional auto-regressive structure is used to model spatial dependence in the Besag ICAR prior. Specifically, it is modelled that the prior distribution of each observation is conditional on the values of its neighbours. A Gaussian distribution is commonly used to describe this conditional dependence. The variance of the distribution represents the degree of spatial autocorrelation, and the mean is a weighted average of the nearby values.

Mathematically, if we denote the observation at location i as $y_{i}$ and its neighbours as $N_{i}$, the Besag ICAR prior can be expressed as:

(13) $$y_{i}|y,N_{i}∼Normal\left(\frac{\sum_{\;j\in N_{i}}w_{i}y_{i}}{\sum_{\;j\in N_{i}}y_{i}},\gamma^{2}\right)$$

where $w_{i}$represents the weight assigned to the relationship between locations i and j, typically based on contiguity or distance, $\gamma^{2}$ represents the precision parameter that controls the degree of spatial autocorrelation (30).

The ability of the Besag ICAR prior to effectively capture spatial dependence without over-fitting the data is one of its main merits. The smoothness constraint it imposes on the geographical effects helps to stabilise the results and prevents the model from attributing too much variability to particular regions.

### 2.5 The independently and identically distributed (IID) assumption

An independent and identically distributed (IID) assumption states that the random effects associated with different clusters are independently drawn from the same distribution and are identically distributed when random effects are added to account for clustered or grouped data in statistical models. For example, an IID assumption for the random effect associated with household numbers implies that the effects of household numbers on AFSD are independently drawn from the same distribution and have the same variability. This is relevant in a study examining the determinants of AFSD from different household numbers, where each household number represents a cluster.

Mathematically, if $u_{1},u_{2},\ldots ,u_{n}$ represent the random effects associated with clusters (e.g., household number) 1,2,…,n, an IID prior for these random effects could be represented as: $u_{i}∼Normal(0,\sigma^{2})$. Here, each $u_{i}$ follows a normal distribution with mean 0 and variance $\sigma^{2}$ and these random effects are assumed to be independent of each other. This assumption simplifies the modelling process, particularly in Bayesian hierarchical models, where it allows us to specify a single prior distribution for the random effects across all clusters (31).

A more explicit representation of the data structure on the model is:

(14) $$\begin{array}{rl} \eta_{\mathrm{AFSD}} & =f_{0}(t)+HHN_{\mathrm{iid}}+AHH\beta +SHH\beta_{1}+EM\beta_{2}+RSA\beta_{3}\\ & +HEL\beta_{4}+IU\beta_{5}+WID\beta_{6}+religion\beta_{7}+TPR\beta_{8}\\ & +region\beta_{9}+f_{spatial(B.ICAR)}(state) \end{array}$$

where β is the effect of the metric covariate AHH, $\beta_{i}$’s denote the linear predictors for the respective categorical covariates, for i=1,…,9, $f_{spat}(s_{i})$ represents a structured geographical frailty effect for unobserved heterogeneity across states in Nigeria and $HHN_{iid}$ also represents the structured frailty effect for unobserved heterogeneity across households.

## 3 Results

From Table 2 it can be seen that the covariates SHH and TPR failed the proportional hazards assumption with p-values below the 0.05 threshold, the global test also failed the test, suggesting the choice of an accelerated failure time modelling strategy for the survival data.

**Table 2:** Constant hazard postulation.

Covariates	Chisq	d.f	*p*-value
AAH	10.651	1	0.0011
SHH	0.161	1	0.6878
EM	32.175	2	$1.0\times 10^{-07}$
RSA	19.406	2	$6.1\times 10^{-05}$
HEL	88.532	3	$2\times 10^{-16}$
UI	50.5529	2	$1.1\times 10^{-11}$
WID	37.434	4	$1.5\times 10^{-07}$
Religion	24.353	4	$6.8\times 10^{-05}$
TPR	1.955	1	0.1620
Region	162.949	5	$2\times 10^{-16}$
Global	456.390	25	$2\times 10^{-16}$

We evaluate the performance of different models and select the one that best fits the data. This selection is based on three criteria: Deviation Information Criterion (DIC) and Watanabe Akaike Information Criterion (WAIC). The model with the lowest values for all three criteria is considered to be the best fit to the data set (see Table 3).

**Table 3:** Model selection for AAFSD analysis.

Model	DIC	WAIC
No clustering	744804.33	744845.55
IID clustering	742633.81	742811.97
Besag ICAR	739215.71	739302.12
IID and besag ICAR	737085.67*	737308.05*

* Indicates the models that best represent for the simulated models considering the DICs and WAICs criteria.

Table 3 shows that the multi-cluster random effects model emerges as the superior choice for analysing Adolescent First Sexual Debut (AAFSD) data. This model outperforms all other options, including those with no random effects, those with only IID random effects (ignoring Besag-ICAR), and those with only Besag-ICAR random effects (ignoring IID). Its dominance is demonstrated by the lowest DIC and WAIC values, indicating better predictive power. The analysis also shows that the introduction of random effects significantly improves the model performance compared to a model without random effects. Furthermore, the model incorporating spatial effects via Besag-ICAR outperforms the model capturing shared unobserved heterogeneity between family members via the IID Gaussian prior.

**Figure 1:**
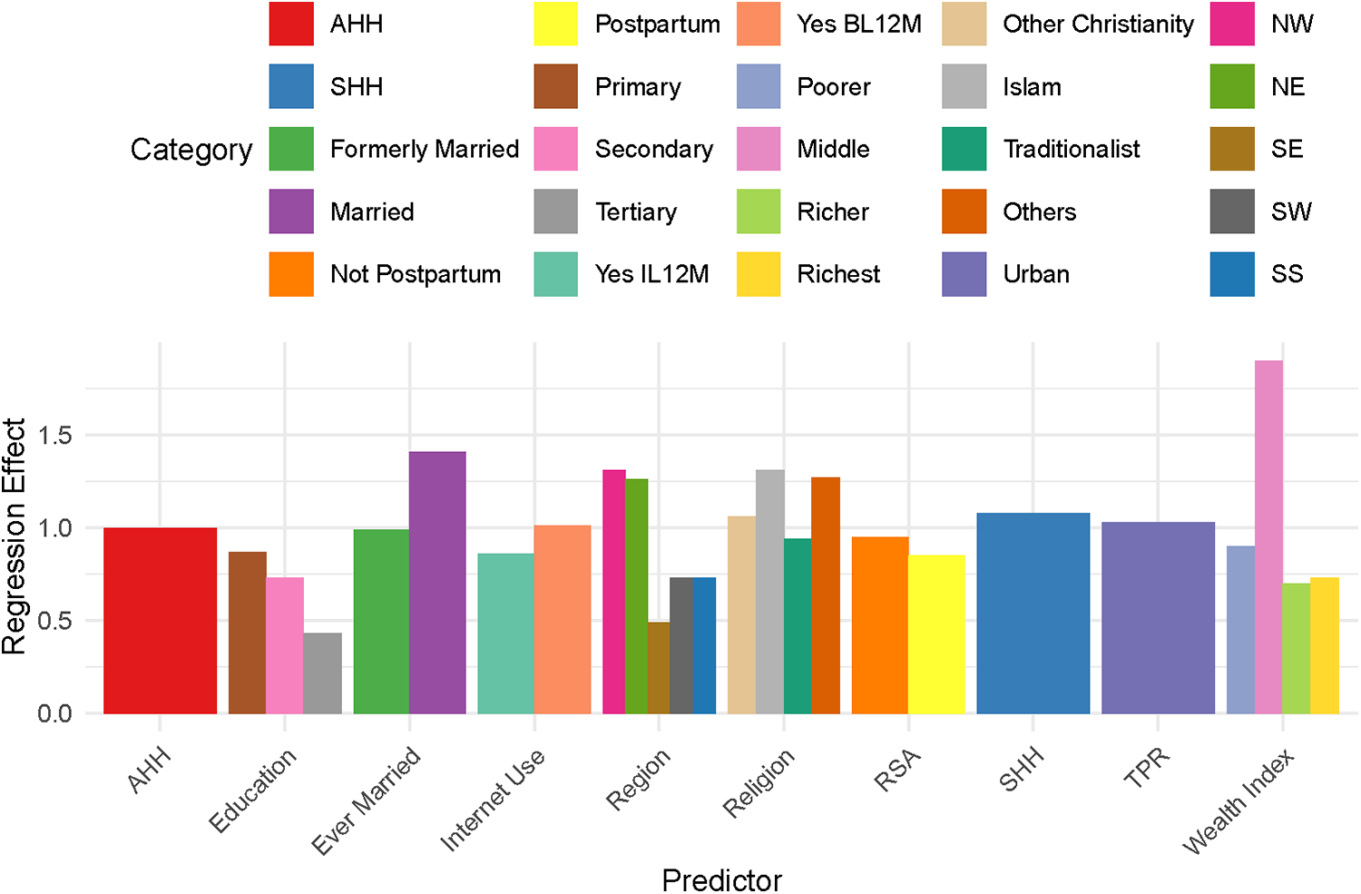
Multiple bar chart of regression effects.

The values estimated for each factor in the model represent the change in time to the event (first sexual debut) compared to a reference category, taking into account random clustering effects. Each coefficient represents the difference in survival time between a given category and the reference category, taking into account the influence of other covariates, IID random clustering and spatial dependencies modelled by the Besag ICAR prior (Table 4, Figure 1).

**Table 4:** Fixed effects estimates and time ratios for AAFSD multi-cluster model.

Fixed effects	Classification	Posterior means	Weibull time ratio
(Intercept)		2.895	
AHH		−0.003	0.997
SHH	Male	0.075	1.08
Ever married	Formerly (1)	−0.012	0.99
	Married (2)	0.343	1.41
Resent sexual activity	Not postpartum	−0.05	0.95
	Postpartum	−0.158	0.85
Education	Primary	−0.136	0.87
	Secondary	−0.317	0.73
	Higher	−0.843	0.43
Internet use	Yes, in the last 12 months	−0.154	0.86
	Yes, before the last 12 months	0.012	1.01
Wealth index	Poorer	−0.11	0.896
	Middle	−0.1	0.90
	Richer	−0.352	0.70
	Richest	−0.321	0.73
Religion	Other christians	0.06	1.06
	Islam	0.268	1.31
	Traditionalist	−0.058	0.94
	Others	0.24	1.27
TPR	Urban	0.028	1.03
Region	NE	0.267	1.31
	NW	0.233	1.26
	SE	−0.71	0.49
	SS	−0.319	0.73
	SW	−0.317	0.73

The estimated coefficient of AHH is −0.003 and the time ratio (TR) is approximately 1.00, representing respectively a slight delay in survival time for a unit increase in AHH and a TR of 1.00 indicating no change in survival time with respect to AHH.

Male sex and living in an urban area have a positive effect on AFSD survival time, accelerating it by 8% and 3% respectively, compared to female sex and those living in the rural areas. Formerly married and married at the time of the survey have a negative and positive effect on AFSD survival time, respectively decelerating it by 1% and accelerating it by 41% compared to the reference category “Not married.” Not postpartum and postpartum both have a negative effect on AFSD survival time, decreasing it by 5% and 15%, respectively, compared to those with no record of recent sexual activity. Primary, secondary and tertiary education all have a negative effect on AFSD survival time, decelerating it by 13%, 27% and 57% respectively, compared to those with no education.

For internet use (IU), those who have used the internet in the last 12 months (UIL12Ms) and those who have used the internet before the last 12 months (UIBL12Ms) have a negative and positive effect on AFSD survival time, respectively decelerating and accelerating AFSD by 14% and 1% compared to the reference of “none.”

The “poorer,” “middle,” “richer” and “richest” categories all have a negative effect on AFSD survival time, delaying it by 10%, 10%, 30% and 27% compared to the “poorest” category.

“Other Christian,” “Islam” and “Others” have positive effects on the survival time of AFSD, increasing it by 6%, 31% and 27% respectively. The “Traditionalist” has a negative effect on survival time, causing an early FSD before the age of 18 years, compared to “Catholic” (reference), respectively.

The “North East” and “North West” both have a positive effect on AFSD, increasing survival time by 31% and 26% respectively, while the “South East,” “South South,” “South West” have a negative effect on the survival time of experiencing FSD, delaying the survival time to experience FSD before the age of 18 by 51%, 27% and 27% respectively, when compared to those from the “North Central.”

**Figure 2:**
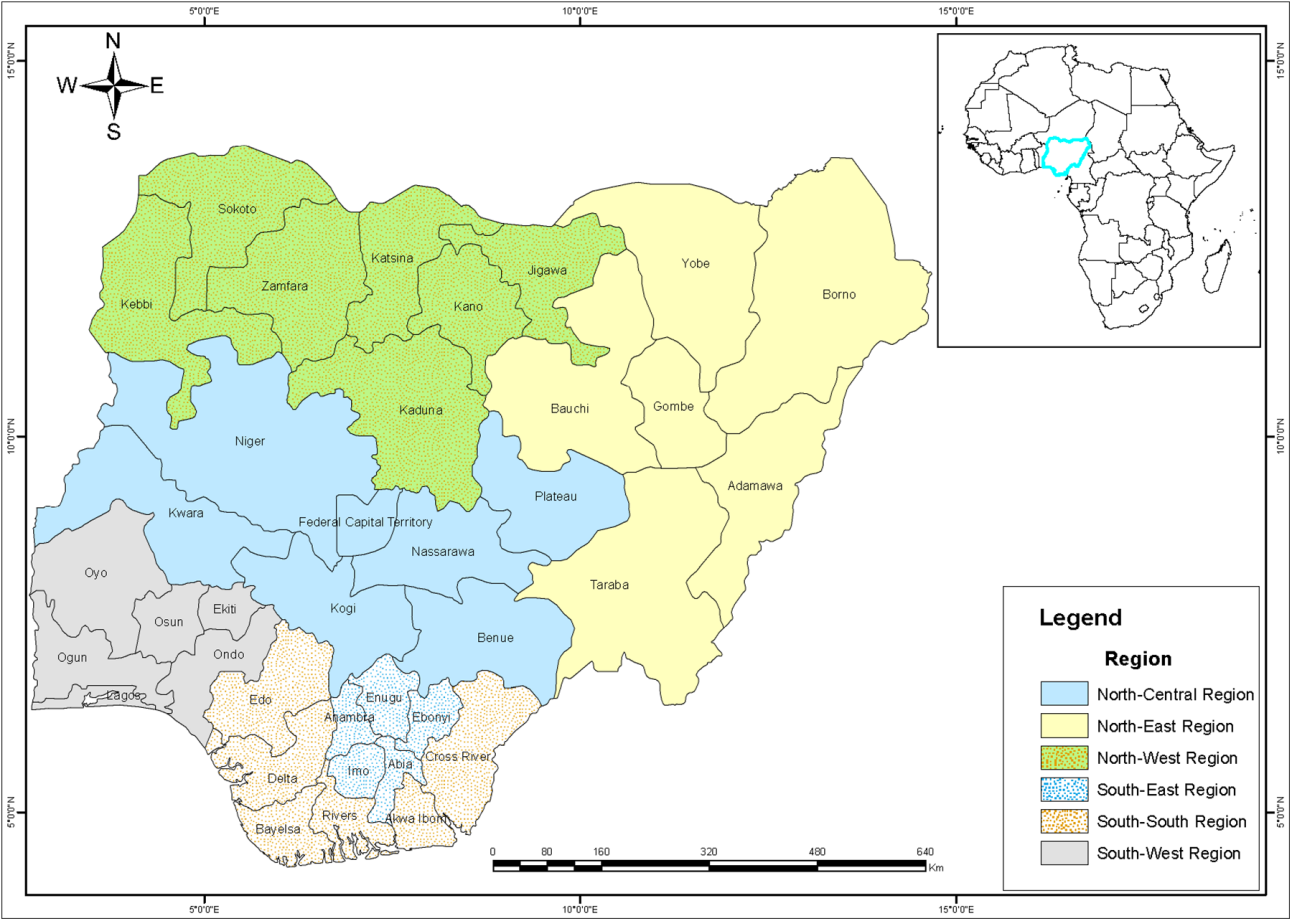
Spatial distribution of states and region in Nigeria.

**Figure 3:**
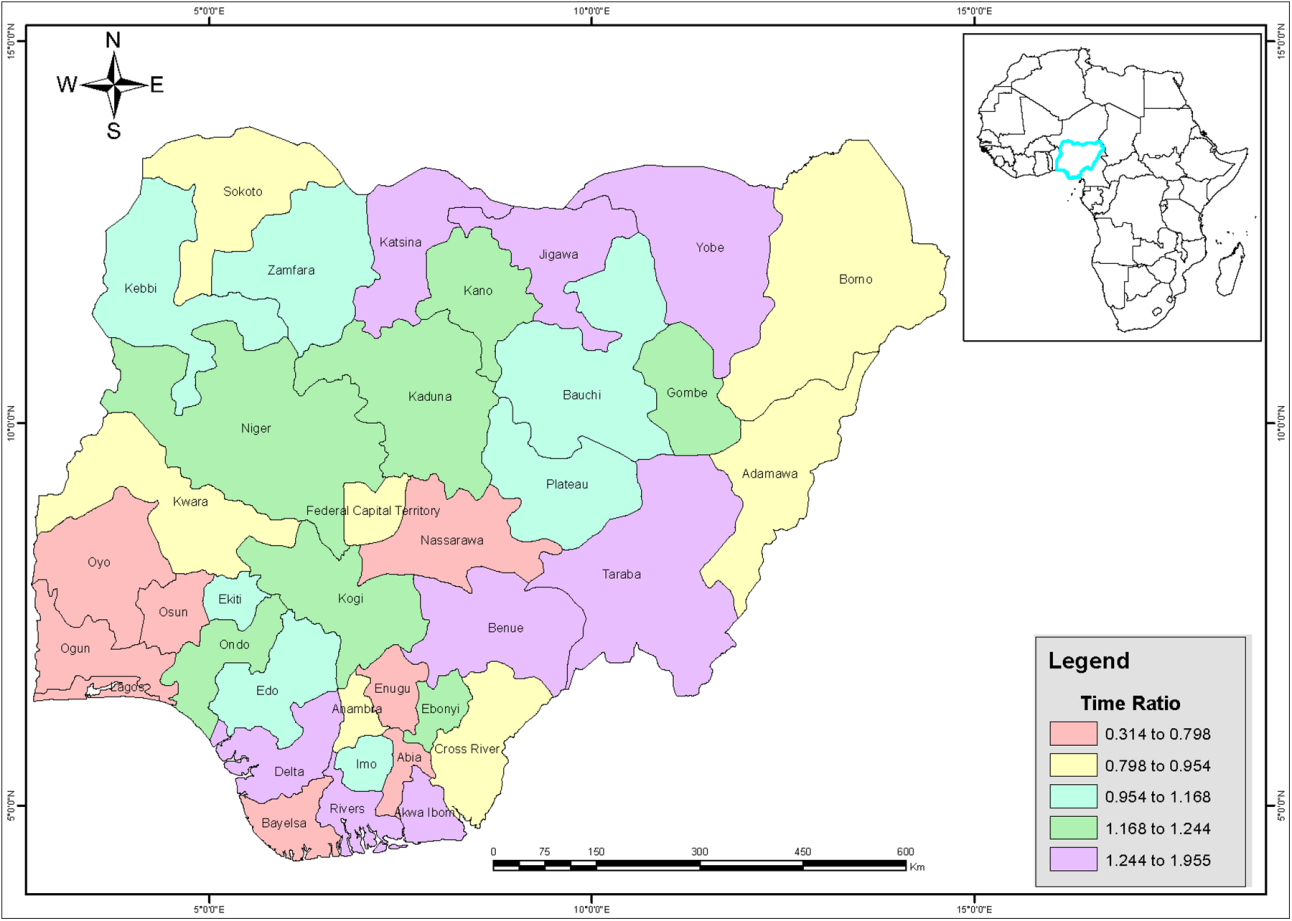
Mapping AFSD risk in Nigeria using the continuous time ratio method.

Again, it is worth noting that the estimates for all covariates included in the model, the IID for HHN accounting for random clustering effects for unobserved heterogeneity between HHNs all contribute and provide the mean posterior values modelled and visualised on the Nigeria maps (Figures 2, 3) for spatial dependencies through the Besag ICAR prior.

Figures 2, 3 show the posterior effects on states. States such as Bayelsa, Abia, Enugu, Nasarawa, Oyo, Ogun Lagos, Osun, with the deep green area have decelerating survival time between 79% and about 20% of residence in these states to experience FSD before their eighteenth birthday, these were mainly observed across the southern region below the Niger. States such as Kwara, Anambra, Cross River, Adamawa, Borno, Sokoto and the FCT, which are shaded in a fairly light green, have between 20% and 5% lower odds of experiencing FSD before their eighteenth birthday. Imo, Edo, Ekiti and the northern states of Plateau and Bauchi, shaded white, have a median accelerated survival time of about 6% of experiencing FSD before their eighteenth birthday. Eboyi, Ondo, Kogi, Niger, Kaduna, Kano, Gombe shaded brown have an accelerated survival time of between 17% and about 24% of living in these states to experience FSD before their eighteenth birthday. States with red colour like; Delta, Rivers AkwaI-bom, Benue, Taraba, Jigawa, Yobe and Kastina have accelerated survival time between 24% to about 95% of residence in these states to experience FSD before their eighteenth birthday.

## 4 Discussion

This paper investigated FSD in Nigeria, using secondary data from the National Demographic Health Survey (NDHS) and setting up a survival analysis layout, defining an event as “1” if age at FSD was less than or equal to 17 years and “0” otherwise. Most covariates, including the global test, were found to violate the proportional hazards assumption for the Cox model—suggesting an alternative survival modelling strategy using the Accelerated Failure Time (AFT) Weibull baseline for Bayesian estimation.

More interestingly, the study proposes a multi-cluster survival effect model—a model that incorporates both independent and identically distributed (IID) and Besag intrinsically conditional autoregressive (ICAR) random effect priors for age at first sexual debut in Nigeria using a generalised additive model. Three existing models were compared with the proposed model, a model with no cluster random effect, a model without Besag (ICAR) and including IID, and a model without IID and including only Besag ICAR.

The result showed that the models with cluster random effect were better than the situation where the clustering effects was ignored, which is consistent with the opinion of (21). However, the proposed ensemble clustering effect of Besag ICAR and IID model outperformed the existing ones with the lowest values of deviance information criterion (DIC) and widely applicable information criterion (WAIC). This suggests that the combination of the two random effect priors captures the complex clustering patterns of age at first sexual debut in Nigeria.

The time ratios for all fixed effect factors were affected by the ensemble clustering effect compared to previous studies; formerly married and married have a 1% deceleration and 41% acceleration in survival time (delay FSD) compared to those “not married,” which is consistent with the finding of (15) who suggested that the unmarried have a higher risk of early sexual debut. Educational levels; “Primary,” “Secondary” and “Higher” indicate a 13%, 27% and 57% of early sexual debut before the age of 18 compared to those with “no education,” which is again compared to the submission of (16) who claimed that FSD is faster among those with no no or lower levels education.

The wealth index also shows that the poor experience FSD more quickly than the rich, as the poorer, middle, rich and richest were observed with a decelerating survival time of 10%,10%,30% and 28% respectively compared to the poorest, which is consistent with the view of (16) who suggested that the poorest are more vulnerable to early sexual initiation. “Internet use, with a 14% decrease and a 1% increase in survival to FSD for those who used the internet in the last 12 months and before the last 12 months, respectively, compared with those who did not use the internet; this implies that used internet most recently within the last 12 months when the survey was conducted will have an early sexual debut. Other Christians, Islam and other religions have an accelerated survival time of 6%, 31% and 27% respectively, while Traditionalists have a decelerated survival time to FSD (early sexual debut) before their eighteenth birthday compared to Catholics. Urban settlers have a 3% accelerated survival time (delayed FSD) than those in rural areas, his is again seen to be consistent with the submissions of (14–18).

The other northern regions were observed to have a decelerating survival time, while the southern regions had an accelerated survival time compared to the north-central regions.

The results of the Nigerian map visualisation revealed distinct clusters of early sexual debut, with significant geographical and socio-demographic variation, identifying states such as Delta, Rivers Akwa-Ibom, Benue, Taraba, Jigawa, Yobe and Kastina as having accelerated survival times between 24% and about 95% for experiencing FSD before their eighteenth birthday.

The statistical findings reveal significant regional disparities in adolescent sexual debut across Nigeria, with notable variations in the median age of first sexual intercourse. These findings have profound societal, cultural, and policy implications.

• Socio-economic Factors: Poverty, limited access to education, and economic disparities can significantly influence adolescent sexual behavior. In regions with higher poverty rates and limited educational opportunities, young people may be more vulnerable to early sexual debut due to factors such as economic pressures, lack of access to information and services, and limited future prospects.
• Cultural Norms: Cultural norms and beliefs regarding sexuality, gender roles, and marriage can also play a crucial role. In some regions, early marriage and childbearing may be socially accepted or even encouraged, while in others, premarital sex may be strongly stigmatized. These cultural norms can influence adolescent decision-making and shape their sexual behaviors.
• Policy Implications: The findings underscore the need for targeted interventions tailored to the specific socio-economic and cultural contexts of different regions. These interventions could include comprehensive sexuality education programs that address issues such as consent, contraception, and sexually transmitted infections. Additionally, efforts to improve access to education, reduce poverty, and empower young people can contribute to delaying sexual debut and promoting healthier sexual behaviors.

The study identified several key risk factors for early sexual debut among Nigerian adolescents. These include marriage, especially for females; lower levels of education; poverty; and living in certain regions of the country, especially the north.

## 5 Conclusions

This study used survival analysis with bayesian approach techniques to assess the impact of various factors on age at first sexual debut (AFSD) in Nigeria, focusing on the additive survival cluster ensemble effect of IID and Besag ICAR. The results show that the new ensemble model significantly outperforms existing models. The research revealed that factors such as wealth and education delay AFSD, while spatial variation across Nigeria was also observed. Some areas in the south and north show an increased risk of early sexual debut. These spatial differences highlight the need for region-specific interventions to address AFSD in Nigeria. Notably, states in southern Nigeria, such as Bayelsa, Abia, and Enugu, exhibit a later median age of first sexual intercourse compared to states in the north, particularly those in the north-west, like Kaduna and Kano. These findings underscore the need for targeted interventions tailored to the specific socio-economic, cultural, and geographic contexts of different regions. This results suggests two approaches to addressing age at first sexual debut (AFSD) in Nigeria. First, regionally tailored intervention programmes are critical. These programmes should be designed taking into account the specific cultural, socio-economic and geographical factors that influence early sexual behaviour in different areas. This targeted approach may be more effective than a one-size-fits-all strategy. Second, continued research is essential. A deeper understanding of the social, economic and cultural factors that contribute to early sexual initiation is needed. Ongoing monitoring and evaluation of intervention programmes is also essential to assess their effectiveness and make adjustments where necessary. Through continuous learning and adaptation, a more comprehensive strategy to address AFSD in Nigeria can be developed.

### 5.1 Strenghth of the study

This study makes significant contributions to the understanding of factors influencing age at first sexual debut (AFSD) among Nigerian adolescents. Specifically, it introduces a novel multi-clustering survival model that incorporates both individual-level and spatial clustering effects, allowing for a more nuanced and accurate assessment of AFSD. The study also utilizes a large, nationally representative dataset and employs advanced statistical techniques to enhance the reliability and generalizability of the findings. In addition, the spatial analysis provides valuable insights into regional variations in AFSD, highlighting the need for targeted interventions in different parts of the country.

### 5.2 Limitation of the study

This study has several limitations. First, it relies on self-reported data from the 2018 Nigeria Demographic and Health Survey (NDHS), which may be subject to biases such as social desirability and may not fully capture the dynamic nature of sexual behavior. Second, the model may not account for all potential confounding factors that could influence AFSD, such as peer pressure, exposure to sexually explicit content, and mental health. Finally, while the study provides valuable insights into the Nigerian context, the findings may not be fully generalizable to other populations or contexts due to the unique socio-cultural and demographic characteristics of Nigeria.

## Data Availability

The datasets presented in this article are not readily available because data may be made available upon reasonable request. Requests to access the datasets should be directed to Braimah Odunayo, braimahjosephodunayo@aauekpoma.edu.ng.
